# Functional intronic *ERCC1* polymorphism from regulomeDB can predict survival in lung cancer after surgery

**DOI:** 10.18632/oncotarget.4083

**Published:** 2015-05-27

**Authors:** Shin Yup Lee, Mi Jeong Hong, Hyo-Sung Jeon, Yi Young Choi, Jin Eun Choi, Hyo-Gyoung Kang, Deuk Kju Jung, Chengcheng Jin, Sook Kyung Do, Seung Soo Yoo, Yangki Seok, Eung Bae Lee, Kyung Min Shin, Ji Yun Jeong, Won Kee Lee, Jaehee Lee, Seung Ick Cha, Chang Ho Kim, Young Tae Kim, Sanghoon Jheon, Jae Yong Park

**Affiliations:** ^1^ Department of Internal Medicine, School of Medicine, Kyungpook National University, Daegu, Republic of Korea; ^2^ Lung Cancer Center, Kyungpook National University Medical Center, Daegu, Republic of Korea; ^3^ Department of Biochemistry and Department of Cell Biology, and Kyungpook National University, Daegu, Republic of Korea; ^4^ Cell and Matrix Research Institute, Kyungpook National University, Daegu, Republic of Korea; ^5^ Thoracic Surgery, Kyungpook National University, Daegu, Republic of Korea; ^6^ Radiology, Kyungpook National University, Daegu, Republic of Korea; ^7^ Pathology, Kyungpook National University, Daegu, Republic of Korea; ^8^ Biostatistics Center, School of Medicine, Kyungpook National University, Daegu, Republic of Korea; ^9^ Department of Thoracic and Cardiovascular Surgery, Seoul National University School of Medicine, Seoul, Republic of Korea; ^10^ BK21 Plus KNU Biomedical Convergence Program, Department of Biomedical Science, Kyungpook National University, Daegu, Republic of Korea

**Keywords:** ERCC1, polymorphisms, regulomeDB, lung cancer

## Abstract

We searched for potential regulatory single nucleotide polymorphisms (SNPs) in *excision repair cross-complementing group 1* (*ERCC1*) using RegulomeDB, a database integrating information from the Encyclopedia of DNA Elements (ENCODE) project, and investigated their association with survival after surgery in non-small cell lung cancer (NSCLC). Among 364 SNPs found within *ERCC1* region using RegulomeDB, four top priority SNPs (rs2298881C>A, rs1049739A>G, rs10415949A>G and rs6509214G>T) were selected for this study. The four SNPs were investigated in 316 patients. A replication study was performed (*n* = 579). Of the four SNPs analyzed in the discovery set, rs2298881C>A and rs6509214G>T were significantly associated with survival outcomes. The association was consistently observed only for rs2298881C>A in the validation cohort. In combined analysis, rs2298881C>A was significantly associated with worse overall survival and disease-free survival (*P* = 0.0002 and 0.02, respectively). A decreased reporter gene expression for rs2298881 A allele was observed compared with C allele by luciferase assay (*P* = 0.02). *ERCC1* rs2298881C>A, an intronic SNP, is the first genetic polymorphism with functional evidence of regulating its expression, and the SNP is associated with prognosis of NSCLC. Our result supports the role of RegulomeDB as a comprehensive source of prioritized candidate SNPs for genetic association studies.

## INTRODUCTION

Excision repair cross-complementing group 1 (ERCC1) is involved in nucleotide excision repair pathway that eliminates bulky DNA adducts caused by carcinogens in tobacco smoke and platinum-based chemotherapeutic agents [1, 2]. Therefore, ERCC1 has been linked to protection against development and progression of cancer, and resistance to platinum-based anticancer drugs at the same time: the double-edged sword. Based on the biological significance of ERCC1, its use as a predictive or prognostic biomarker has been pursued by a large number of cancer researchers. The expression of *ERCC1* by quantitative real-time polymerase chain reaction or immunohistochemistry has been correlated with the clinical outcomes of non-small cell lung cancer (NSCLC) [3–5]. Genetic polymorphism of *ERCC1* has also been investigated for the association with the risk and clinical outcome of many types of cancer including NSCLC [6–14]. The most widely studied single nucleotide polymorphisms (SNPs) include rs11615T>C (N118N) which is the only SNP tested in the exon region of *ERCC1*, and rs3212986C>A in 3′- UTR of *ERCC1* (Q504K for *CD3EAP*, antisense to *ERCC1*). Not surprisingly, the association of these SNPs with NSCLC has not been consistent across studies.

The human genome project has revealed that only 2% of human genome contains protein-coding genes, with the vast majority of human genome remained as ‘junk DNA’ [15]. However, despite intensive studies focused on protein-coding genes, our understanding of the genome has been far from complete [16]. In addition, nearly 90% of the variants identified as phenotype-associated SNPs in genome-wide association studies (GWAS) have been located within intergenic or intronic regions, posing an obstacle to its interpretation [16, 17]. Therefore, it has been suggested that the genome region outside the protein-coding genes may have the key to open the treasure chest of the vast genetic information of human genome.

The Encyclopedia of DNA Elements (ENCODE) project has the aim of describing all functional elements encoded in the human genome [16]. It revealed that 80% of the genome, especially outside of protein-coding regions, contains elements linked to biochemical functions such as DNA-transcription factor binding, providing new insights into the mechanisms of gene regulation [16]. RegulomeDB is a database which integrates a large collection of regulatory information from ENCODE and other data sources [17], being a rich source of information that may provide putative mechanistic explanations for genetic association studies including GWAS [18]. Until recently, a few studies utilized RegulomeDB to predict regulatory function of SNPs in non-coding regions that were identified by GWAS or candidate gene study [19–22].

RegulomeDB provides a scoring system prioritizing SNPs based on the degree of experimental or computational evidence that a variant lies in a functional location and likely results in a functional consequence, e.g., alteration of transcription factor binding and gene expression [17]. Therefore, selection of potential regulatory SNPs using RegulomeDB may help to improve power to detect true causal variants in genetic association studies. In the present study, we selected SNPs with high confidence of functional consequence in *ERCC1* gene region using RegulomeDB and investigated the association between those SNPs and the survival of NSCLC patients after curative surgery.

## RESULTS

### Patient characteristics and clinical predictors

The clinical and pathologic characteristics of patients in the discovery and validation sets and the association with OS and DFS are shown in Table 1. Upon univariate analysis, pathologic stage was significantly associated with OS and DFS in both sets (log-rank *P* [*P*_L-R_] for OS = 2.0 × 10^−6^ and 0.0006; and *P*_L-R_ for DFS = 4.0 × 10^−10^, 5.0 × 10^−7^, respectively). Gender was associated with OS in the discovery set (*P*_L-R_ for OS = 0.04), and age was associated with OS and DFS in the validation set (*P*_L-R_ for DFS = 0.0001 and 0.01, respectively).

**Table 1:** Univariate analysis for overall survival and disease-free survival by clinicopathological characteristics in the discovery cohorts and validation cohorts

Variables	No. of cases	Discovery set						No. of cases	Validation set					
		Overall survival			Disease-free survival				Overall survival			Disease-free survival		
		No. of death (%)*	5Y-OSR (%)^†^	Log-Rank *P*	No. of event (%)*	5Y-DFSR (%)^†^	Log-Rank *P*		No. of death (%)*	5Y-OSR (%)^†^	Log-Rank *P*	No. of event (%)*	5Y-DFSR (%)^†^	Log-Rank *P*
**Overall**	316	126 (39.9)	53		160 (50.6)	43		579	164 (28.3)	67		296 (51.1)	45	
**Age (years)**														
≤63	161	60 (37.3)	57	0.1	81 (50.3)	44	0.59	306	70 (22.9)	74	0.0001	146 (47.7)	50	0.01
> 63	155	66 (42.6)	48		79 (51.0)	41		273	94 (34.4)	58		150 (55.0)	38	
**Gender**														
Female	74	19 (25.7)	62	0.04	33 (44.6)	45	0.63	164	39 (23.8)	73	0.08	81 (49.4)	46	0.54
Male	242	107 (44.2)	50		127 (52.5)	42		415	125 (30.1)	65		215 (51.8)	45	
**Smoking status**														
Never	72	21 (29.2)	63	0.09	34 (47.2)	43	0.73	202	55 (27.2)	69	0.37	109 (54.0)	40	0.61
Ever	244	105 (43.0)	50		126 (51.6)	43		377	109 (28.9)	66		187 (49.6)	48	
**Pack-years^‡^**														
< 40	106	43 (40.6)	51	0.37	75 (51.4)	42	0.80	175	47 (26.9)	68	0.18	85 (48.6)	42	0.37
≥ 40	138	62 (44.9)	49		51 (52.0)	43		202	62 (30.7)	64		102 (50.5)	47	
**Histological type**														
SCC	172	67 (39.0)	54	0.83	81 (47.1)	46	0.34	241	67 (27.8)	68	0.18	111 (46.1)	51	0.16
AC	139	56 (40.3)	51		76 (54.7)	37		312	86 (27.6)	68		169 (54.2)	41	
LCC	5	3 (60.0)	60		3 (60.0)	60		26	11 (42.3)	52		16 (61.5)	42	
**Pathologic stage**														
I	194	55 (28.4)	61	2.0 × 10^−6^	72 (37.1)	53	4.0 × 10^−10^	348	82 (23.6)	71	0.0006	152 (43.7)	51	5.0 × 10^−7^
II+IIIA	122	71 (58.2)	40		88 (72.1)	27		231	82 (35.5)	61		144 (62.3)	36	
**Adjuvant therapy**														
No	67	37 (55.2)	39	0.79	47 (70.1)	29	0.56	110	40 (36.4)	65	0.87	72 (65.5)	37	0.75
Yes	55	34 (61.8)	41		41 (74.6)	26		121	42 (34.7)	56		72 (59.5)	34	

Abbreviations: SCC, Squamous cell carcinoma; AC, adenocarcinoma; LCC, Large cell carcinoma.

* Row percentage.

† Five year-overall survival rate (5Y-OSR) and 5 year-disease free survival rate (5Y-DFSR), proportion of survival derived from Kaplan-Meier analysis.

‡ In ever-smokers.

### Associations between SNPs and survival outcomes

**Figure 1:**
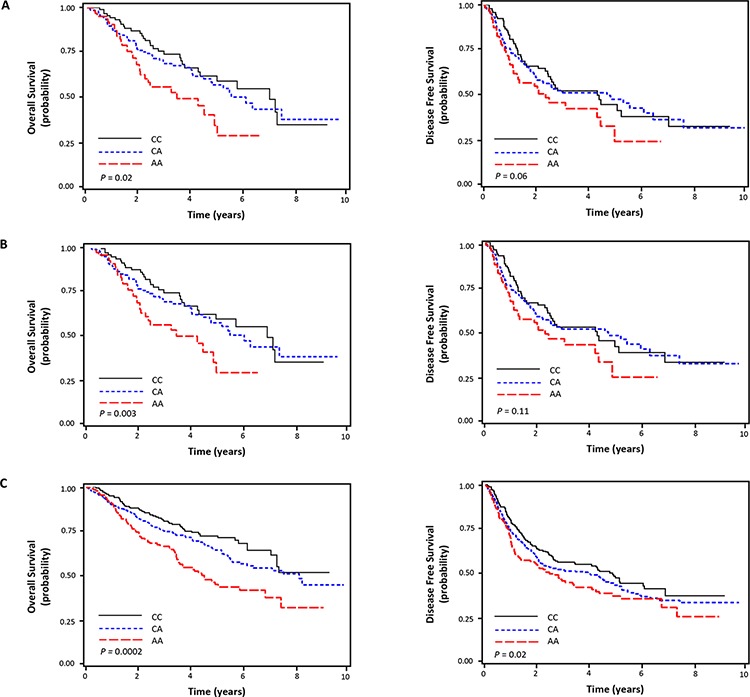
Kaplan-Meier plot of overall and disease free survival curves according to *ERCC1* rs2298881C > A genotype in discovery cohort **A.** replication cohort **B.** and combined cohort **C.** *P* values in the multivariate Cox proportional hazard model.

Among the four SNPs analyzed in the discovery set, the rs2298881C>A and rs6509214G>T were significantly associated with survival outcomes when adjusted for age, gender, smoking status, tumor histology, pathologic stage, and adjuvant therapy (Table 2). However, the association was consistently observed only for the rs2298881C > A in an independent validation set, which was in the same direction as the discovery set. In combined analysis, the rs2298881C > A was significantly associated with worse OS and DFS (adjusted HR [aHR] for OS, 1.37; 95% CI, 1.16–1.63; *P* = 0.0002; aHR for DFS, 1.17; 95% CI, 1.03–1.34; *P* = 0.02; under additive genetic model; Table 2 and Figure 1).

**Table 2:** Association of rs2298881C>A and rs6519214G>T and survival outcomes in the discovery and validation sets

ID No.*	Genotypes	Discovery cohort				Validation cohort				Combined cohort			
		Overall survival		Disease-free survival		Overall survival		Disease-free survival		Overall survival		Disease-free survival	
		HR (95%CI)^†^	*P*^†^	HR (95%CI)^†^	*P*^†^	HR (95%CI)^†^	*P*^†^	HR (95%CI)^†^	*P*^†^	HR (95%CI)^†^	*P*^†^	HR (95%CI)^†^	*P*^†^
rs2298881^‡^	CC	1.00		1.00		1.00		1.00		1.00		1.00	
	CA	1.18 (0.75−1.86)	0.47	1.07 (0.73−1.57)	0.73	1.47 (0.97−2.23)	0.07	1.26 (0.95−1.68)	0.11	1.30 (0.96−1.75)	0.09	1.19 (0.95−1.49)	0.13
	AA	1.92 (1.15−3.20)	0.01	1.59 (1.01−2.48)	0.04	2.00 (1.26−3.16)	0.003	1.31 (0.93−1.84)	0.13	1.88 (1.34−2.63)	0.000	1.38 (1.05−1.80)	0.02
	Dominant	1.37 (0.90−2.09)	0.14	1.20 (0.84−1.72)	0.31	1.63 (1.10−2.42)	0.02	1.28 (0.98−1.67)	0.08	1.46 (1.10−1.94)	0.01	1.24 (1.00−1.54)	0.05
	Recessive	1.73 (1.14−2.63)	0.01	1.52 (1.04−2.22)	0.03	1.55 (1.09−2.19)	0.01	1.12 (0.85−1.49)	0.42	1.58 (1.21−2.06)	0.001	1.23 (0.98−1.54)	0.07
	Additive	1.39 (1.06−1.81)	0.02	1.25 (0.99−1.58)	0.06	1.41 (1.13−1.77)	0.003	1.15 (0.97−1.36)	0.11	1.37 (1.16−1.63)	0.000	1.17 (1.03−1.34)	0.02
rs6509214^§^	GG	1.00		1.00		1.00		1.00		1.00		1.00	
	GT	1.47 (0.97−2.24)	0.07	1.63 (1.13−2.37)	0.01	0.91 (0.60−1.38)	0.65	0.97 (0.71−1.31)	0.82	1.17 (0.87−1.57)	0.30	1.21 (0.95−1.52)	0.12
	TT	1.67 (0.94−2.97)	0.08	1.61 (0.97−2.67)	0.06	0.87 (0.51−1.49)	0.61	0.75 (0.50−1.12)	0.16	1.10 (0.75−1.61)	0.64	0.99 (0.73−1.35)	0.95
	Dominant	1.51 (1.01−2.26)	0.05	1.63 (1.14−2.33)	0.01	0.90 (0.60−1.33)	0.59	0.90 (0.67−1.20)	0.45	1.15 (0.87−1.52)	0.33	1.14 (0.91−1.43)	0.24
	Recessive	1.31 (0.80−2.16)	0.29	1.18 (0.76−1.83)	0.45	0.93 (0.58−1.48)	0.75	0.77 (0.54−1.09)	0.14	1.00 (0.71−1.40)	0.99	0.89 (0.68−1.16)	0.38
	Additive	1.32 (1.01−1.73)	0.05	1.32 (1.04−1.66)	0.02	0.93 (0.72−1.21)	0.59	0.88 (0.73−1.06)	0.19	1.06 (0.89−1.28)	0.52	1.02 (0.88−1.18)	0.78

Abbreviations: HR, hazard ratio; CI, confidence interval.

* Information about polymorphisms and IDs were obtained from NCBI database (http://www.ncbi.nlm.nih.gov/SNP).

† HRs, 95% CIs and their corresponding *P*-values were calculated using multivariate Cox proportional hazard models, adjusted for age, gender, smoking status, tumor histology, pathologic stage, adjuvant therapy.

‡ The numbers of patients in discovery, validation, and combined cohort for rs2298881 were 316, 579, and 895, respectively.

§ The numbers of patients in discovery, validation, and combined cohort for rs6509214 were 316, 412, and 728, respectively, due to lack of available samples for validation.

### Effect of rs2298881C > A on the promoter activity of *ERCC1*

**Figure 2:**
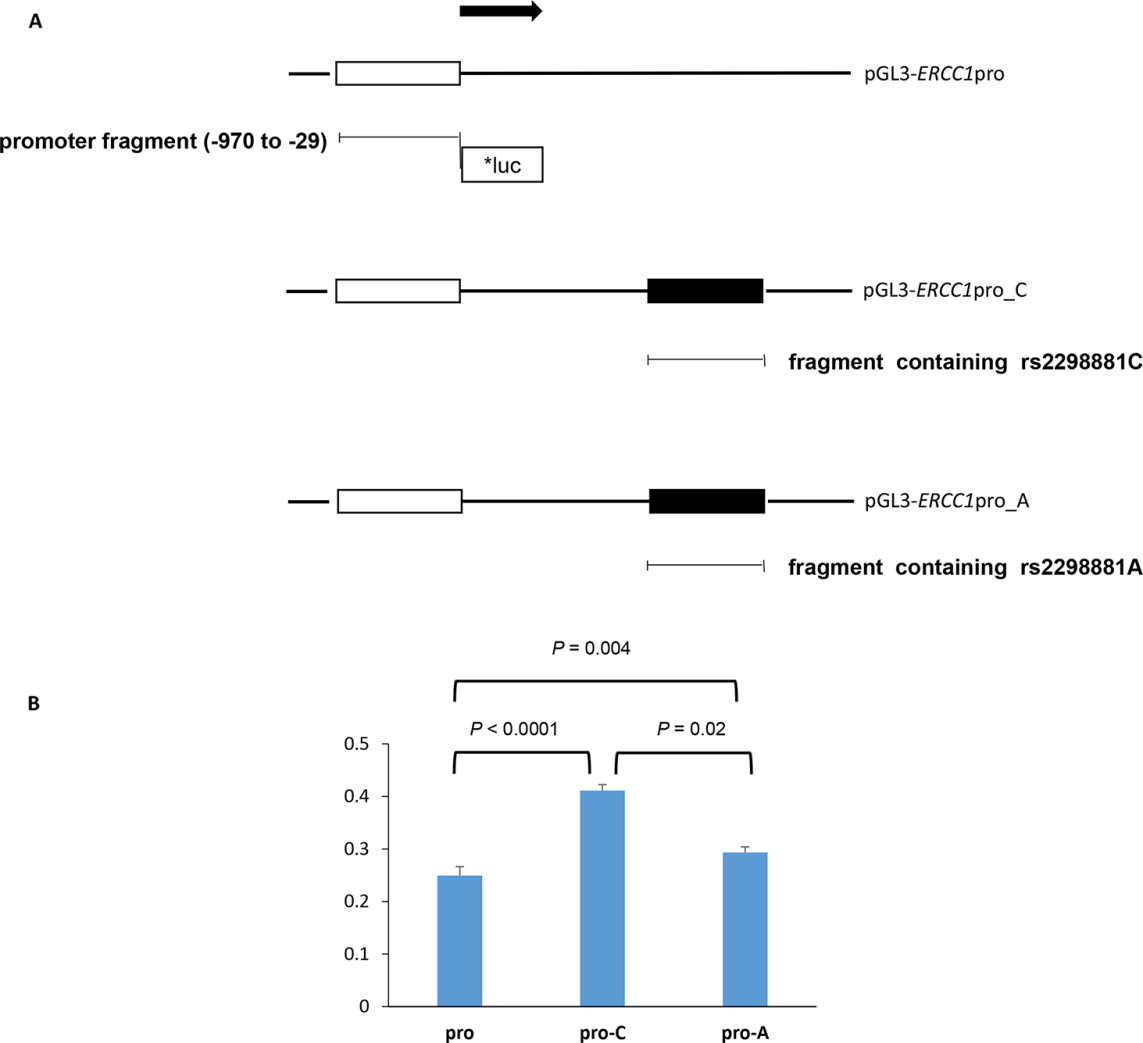
Functional analysis of the *ERCC1* rs2298881C>A **A.** Schematic representation of the constructs that were used for the reporter gene assays. Promoters are marked by white blocks and the fragments including rs2298881C> A site by black blocks, and arrow indicates the direction of transcription. The first base of translation start site is denoted as +1. *ERCC1* promoter was amplified from human genomic DNA and cloned into the pGL3 basic vector (pGL3-*ERCC1*pro). DNA fragments containing the SNP site were cloned into other multi cloning site (pGL3-*ERCC1*pro_C and pGL3-*ERCC1*pro_A). **B.** Luciferase activity according to *ERCC1* rs2298881C>A. H1299 cells were transfected with pGL3-*ERCC1*pro, pGL3-*ERCC1*pro_C and pGL3-*ERCC1*pro_A constructs, respectively. Each bars represent mean ± S.E.M. of firefly luciferase activity normalized to *Renilla* luciferase activity. Experiments were performed in triplicate. *P* value, a Student's *t*-test. luc, luciferase.

To investigate whether rs2298881C > A affects promoter activity of *ERCC1*, we generated three pGL3-*ERCC1* constructs: pGL3-*ERCC1*pro with cloned *ERCC1* promoter region alone, and pGL3-*ERCC1*pro_C and pGL3-*ERCC1*pro_A with both promoter region and the fragment containing rs2298881C>A (Figure 2A). As shown in Figure 2B, luciferase activity was significantly higher in H1299 cells transfected with pGL3-*ERCC1*pro_C or pGL3-*ERCC1*pro_A compared with pGL3-*ERCC1*pro, suggesting that the fragment containing rs2298881C>A enhanced the activity of *ERCC1* promoter. A decreased expression of the reporter gene for the A allele of rs2298881C>A was observed compared with the C allele by luciferase assay (*P* = 0.02; Figure 2B). These results suggest that an intronic SNP rs2298881C>A may alter *ERCC1* expression by affecting *ERCC1* promoter activity.

## DISCUSSION

We investigated the association between potential regulatory SNPs in *ERCC1* gene region selected from RegulomeDB and survival of patients with surgically resected early stage NSCLC in a relatively large two-stage study including 895 patients. Our study showed significant association between *ERCC1* rs2298881C>A and the prognosis of patients with early stage NSCLC, which was reproducible in an independent set of patients. We also report that rs2298881C>A, an intronic SNP of *ERCC1*, is the first genetic polymorphism with functional evidence of regulating *ERCC1* expression. These findings suggest that *ERCC1* rs2298881C>A could be used as a prognostic marker for early stage NSCLC, and that RegulomeDB may be useful in selecting potentially functional SNPs in the regulatory region for genetic association studies.

In the present study, we searched for regulatory SNPs in *ERCC1* gene region using RegulomeDB and showed that rs2298881C>A was associated with worse prognosis of NSCLC patients after curative resection. *In vitro* luciferase assay showed that the *ERCC1* rs2298881C-to-A change was associated with reduced promoter activity of *ERCC1*. According to RegulomeDB, the rs2298881C>A has the highest level of evidence for regulatory role among SNPs in *ERCC1* gene region. In addition, based on RegulomeDB, rs2298881C>A is the only SNP throughout the whole genome reported to be in the eQTL that is predicted to regulate the expression of *ERCC1*. Our result is in line with the realization of regulatory function of non-coding DNA and suggests the need for investigating variants in regulatory region.

Recent results of ENCODE project provided evidence revealing that genetic variation in non-coding DNA play an important role in the regulation of gene expression. The ENCODE data show that the results of GWAS are typically enriched for variants within non-coding functional units, suggesting that many of these regions could be causally linked to disease [17, 18, 24]. Therefore, RegulomeDB containing ENCODE data is a powerful tool for predicting the likelihood of a SNP being in a functional location, thereby facilitates prioritizing SNPs for genetic association studies. The result of our study supports the role of RegulomeDB in selecting putative regulatory SNPs for future genetic association studies.

Genetic polymorphisms of *ERCC1* have been investigated in terms of the risk and the clinical outcomes in many types of cancer including NSCLC [6–14]. However, most of the studies have focused on only a few SNPs, such as *ERCC1* rs11615T>C (N118N) and rs3212986C>A in 3′-UTR, and the results have not been consistent among studies. We previously investigated these two SNPs in terms of the clinical outcomes of early-stage NSCLC after surgery and advanced NSCLC after platinum-based chemotherapy in Koreans [13, 25, 26]. However, neither rs11615T>C nor rs3212986C>A showed significant association with the outcome of NSCLC [13, 25, 26]. In the present study, we searched RegulomeDB for potential regulatory SNPs in *ERCC1*, and investigated their association with survival after surgery in NSCLC. In fact, rs2298881C>A and other intronic SNPs such as rs3212961A>C and rs3212948G>C were selected as haplotype tagging SNPs in previous studies that first evaluated those SNPs [27, 28]. However, a small number of studies on rs2298881C>A with variable number of patients have shown discrepant association with various types of cancer [27, 29–31].

In this study, we included a total of 895 patients which is relatively large for studies on surgically resected NSCLC. The association between *ERCC1* rs2298881C>A and survival outcomes was replicated across both discovery and validation sets of the study, which would largely reduce false positivity [32, 33]. In addition, the association of rs2298881C>A with survival outcome was biologically plausible. It is possible that the *ERCC1* rs2298881 C-to-A change in the putative regulatory region may lead to reduced promoter activity and decreased *ERCC1* expression, resulting in decreased DNA repair capacity and therefore worse disease outcome. However, in our preliminary analysis, significant difference in the relative expression level of *ERCC1* mRNA among genotypes of rs2298881C>A was not observed in either tumor or paired non-malignant lung tissues (Supplementary Figure 1). Future studies are required to understand the biologic mechanism of the observed association between the SNPs and survival outcomes.

In conclusion, this study showed that *ERCC1* rs2298881C>A could predict the survival outcomes of patients with surgically resected early stage NSCLC. RegulomeDB may be useful as a practical tool for selecting potentially functional SNPs in the regulatory region for future genetic association studies.

## MATERIALS AND METHODS

### Study population

The discovery set included 316 patients with pathologic stages I, II, or IIIA (micro-invasive N2) NSCLC who underwent curative surgical resection at Kyungpook National University Hospital (KNUH) between September 1998 and August 2007. Genomic DNA samples from tumor and corresponding non-malignant lung tissue specimens were provided by the National Biobank of Korea - KNUH, which is supported by the Ministry of Health, Welfare and Family Affairs. Written informed consent was obtained from all patients prior to surgery. All materials derived from the National Biobank of Korea - KNUH were obtained under institutional review board-approved protocols. The validation set included 579 patients with pathologic stages I, II, or IIIA NSCLC who underwent curative surgical resection at KNUH (*n* = 99) and Seoul National University Hospital (*n* = 307), Seoul National University Bundang Hospital (*n* = 173). Written informed consent was obtained from all patients before surgery and research protocol was approved by the institutional review board at each hospital. All of the patients included in this study were ethnic Koreans. Patients who underwent chemotherapy or radiotherapy prior to surgery were excluded to avoid the effects on DNA. The pathologic staging of the tumors was determined according to the International System for Staging Lung Cancer [23].

### SNP selection and genotyping

Three hundred and sixty four SNPs were found within *ERCC1* gene region, NC_000019.9 (45910591..45982241, complement) by Genome Reference Consortium Human Build 37 patch release 13 (GRCh37.p13) assembly, using RegulomeDB (http://regulome.stanford.edu). The RegulomeDB provides a scoring system with categories ranging from 1 to 6 based on the degree of experimental or computational evidence of functional consequence of a given variant. Category 1 includes variants that are known expression quantitative trait loci (eQTLs), which have been shown to be associated with expression of target genes, and is further divided into subcategories 1a to 1f. Because the lower score indicates the stronger evidence for a variant to be located in a functional region, a variant scored as 1a most likely affects transcription factor binding and expression of a target gene. We prioritized the 364 SNPs using the RegulomeDB, and selected five SNPs that were classified into category 1: two SNPs (rs2298881C>A and rs1049739A>G) had a score of 1b, and three SNPs had a score of 1f (rs10415949A>G, rs6509214G>T, rs7245548C>T). Among those, rs7245548 was not genotyped because it was in linkage disequilibrium with rs6509214 by HapMap JPT database. Finally, four SNPs (rs2298881C>A, rs1049739A>G, rs10415949A>G, and rs6509214G>T) were selected for genotyping to investigate the relationship with the survival of NSCLC. Genomic DNA was extracted from tissues with QIAamp^®^ genomic DNA kit (Qiagen, Hilden, Germany) according to the manufacturer's protocol. The rs2298881C>A was genotyped using the Taq-Man^®^ assay (Applied Biosystems, Foster City, CA) following the manufacturer's instructions. The Taq-Man probes were predesigned and synthesized by Applied Biosystems. The rs10415949A>G and rs6509214G>T were genotyped using SEQUENOM's MassARRAY^®^ iPLEX assay according to instructions of the manufacturer. Duplicate samples and negative controls were included to ensure accuracy of genotyping. For validation of genotyping, approximately 5% of samples of the cohort were randomly selected to be genotyped again with a restriction fragment length polymorphism assay by a different investigator and the results were 100% concordant.

### Cloning of the luciferase reporter gene and luciferase assay

The rs2298881C>A is an eQTL for *ERCC1* gene located in intron 1. RegulomeDB suggests that the SNP lies in a location which overlaps transcription binding site and regulates gene expression. We investigated whether rs2298881C>A affects *ERCC1* promoter activity by luciferase reporter assay. The pGL3-Basic Vector (Promega, Madison, WI, USA) was used to construct luciferase reporter plasmids using manufacturer's protocols. Briefly, promoter region of *ERCC1* (−970 to −29 bp, the transcriptional start site is designated as +1) was synthesized by polymerase chain reaction from human genomic DNA and cloned into the pGL3-Basic vector to generate pGL3-*ERCC1*pro. Two fragments including rs2298881C or rs2298881A allele of *ERCC1* rs2298881C>A were amplified from genomic DNA sample and the 160 bp products were cloned into pGL3-*ERCC1*pro, respectively. All constructs were verified by direct sequencing before use. Human non-small cell lung cancer cells (H1299) were maintained at 37°C in 5% CO_2_ atmosphere in RPMI-1640 medium containing 10% heat-inactivated fetal bovine serum (FBS). The cells were transfected with 300 ng of each plasmid DNA (pGL3-*ERCC1*pro, pGL3-*ERCC1*pro_C, or pGL3-*ERCC1*pro_A) and 30 ng of pRL-SV40 Vector (Promega, Madison, WI, USA) using Effectene^®^ (Qiagen, Hilden, Germany) according to manufacturer's protocol. Luciferase activity was measured on an Orion L Microplate Luminometer (Berthold Detection Systems GmbH, Pforzheim, Germany) using the Dual-Luciferase^®^ Reporter Assay System (Promega, Madison, WI, USA). Firefly luciferase activity measurements were normalized with respect to pRL-SV40 *Renilla* luciferase activity to correct for variations in transfection efficiency. Each experiment was conducted in triplicate.

### Statistical analysis

Differences in the distribution of genotypes according to the clinicopathologic factors of patients were compared using χ^2^ tests. Hardy-Weinberg equilibrium was tested using a goodness-of-fit χ^2^ test with 1 *degree of freedom*. The genotypes for each SNP were analyzed as a three-group categorical variable, and those were also grouped according to the dominant, recessive and additive model. Overall survival (OS) was measured from the day of surgery to the date of the last follow-up or until the date of death. Disease-free survival (DFS) was calculated from the day of surgery until recurrence or death. The survival estimates were calculated using the Kaplan-Meier method. The difference in OS and DFS according to the SNPs was compared using log-rank tests. Cox's proportional hazard regression model was used for the multivariate survival analyses, and the analyses were always adjusted for age (> 63 years versus ≤ 63), gender (male versus female), smoking status (ever versus never), tumor histology (squamous vs. non-squamous), pathologic stage (II-IIIA versus I), and adjuvant therapy (yes vs. no). The hazard ratio (HR) and 95% confidence interval (CI) were also estimated. A cut-off *p*-value of 0.05 was adopted for all the statistical analyses. The statistical data were obtained using SAS Genetic software (SAS Institute, Cary, NC).

## SUPPLEMENTARY DATA, FIGURE


