# Supplementary material for: Functional intronic ERCC1 polymorphism from regulomeDB can predict survival in lung cancer after surgery
Source: Oncotarget. 2015 May 27;6(27):24522–32. doi: 10.18632/oncotarget.4083 (PMC4695203; doi:10.18632/oncotarget.4083)
Supplement: Supplementary file 1 [file oncotarget-06-24522-s001.pdf]

SUPPLEMENTARY DATA

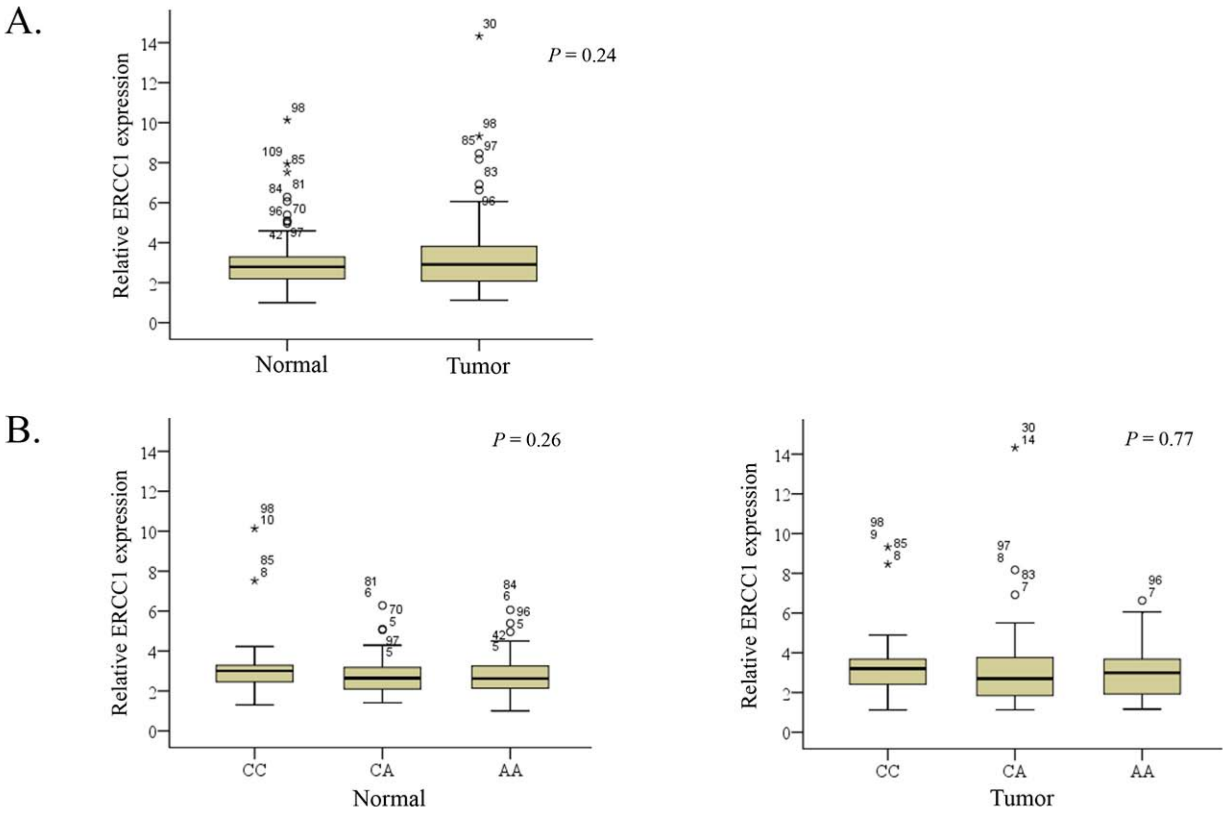

Supplementary Figure S1: *ERCC1* mRNA expression examined by quantitative reverse transcription-polymerase chain reaction (PCR). A. *ERCC1* mRNA expression level in normal lung and tumor tissue. **B.** *ERCC1* mRNA expression level and the association with the rs2298881C > A genotypes (33CC, 48CA, and 25AA) in normal lung and tumor tissue. Total RNA from tumor and paired non-malignant lung tissues (n = 109) was isolated using TRIzol (Invitrogen). Real-time PCR with Taqman detection (Hs01012158_m1 for ERCC1, Hs99999903_m1 for beta-actin) was performed using a LightCycler 480 (Roche Applied Science) with Taqman Gene Expression Master Mix (Applied Biosystems). There were three cases with missing genotypes. Each sample was run in duplicate. The relative *ERCC1* mRNA expression were normalized with beta actin and then calculated by the $2^{-\Delta\Delta Ct}$ method (1).

REFERENCE

1. Livak KJ, Schmittgen TD. Analysis of relative gene expression data using real-time quantitative PCR and the 2(-Delta Delta C(T)) Method. Methods. 2001; 25:402–8.
